# Intraleukocytic Candida is Diagnostic of Pathological Candidemia

**DOI:** 10.5505/tjh.2012.05826

**Published:** 2012-12-05

**Authors:** Anil Handoo, Sarada Nagoti, Adarsh Choudhary

**Affiliations:** 1 Medanta The Medicity, Haematology, Gurgaon, India; 2 Medanta The Medicity, Surgical Gastroenterology, Gurgaon, India

Candidemia is the presence of Candida species in blood. It is the most frequently encountered invasive fungal infection (IFI) in hospitalized patients, and ranks as the fourth most common cause of nosocomial blood stream infection [1]. Despite its high incidence and the availability of a variety of techniques for its detection, the sensitivity of blood cultures for detecting candidemia remains only 50% [2]. As such, candidal bloodstream infections may be vastly under diagnosed and under reported. A number of risk factors associated with candidemia have been identified, including the presence of a central intravenous line, mechanical ventilation, dialysis dependence, major surgery, and use of broad-spectrum antimicrobial agents or total parenteral nutrition [3]. 

Timely diagnosis and initiation of proper antifungal therapy are critical in the management of candidemia and for the prevention of its complications. Most institutions rely on blood cultures alone to diagnose candidemia. Budding yeasts on peripheral blood film are considered a contaminant, whereas their intracytoplasmic presence— in neutrophils or monocytes—should be indicative of the pathological nature of the findings. 

A 16-year-old male undergoing follow-up for polyarteritis nodosa (PAN) was admitted to our hospital with a burst abdomen due to mesenteric ischemia. The patient underwent emergency laparotomy and bowel resection with jejunostomy. The patient developed wound dehiscence 2 d post surgery. Re-exploratory laparotomy with adhesiolysis was performed and broad-spectrum antibiotic treatment was commenced. He later developed fever and bleeding at the stoma site. At this time his peripheral blood findings were as follows: Hb: 7.5 g/dL; WBC: 4.5 x 109/L; neutrophils: 58.8%; lymphocytes: 26.5%; eosinophils: 0.7%; monocytes: 13%; basophils: 1%; platelet count: 87 x 109/L. Peripheral blood smear showed dimorphic anemia and thrombocytopenia, as well as neutrophilic predominance with mild left shift and thrombocytopenia. In addition, the smear showed that there were many extra-leukocytic budding yeasts, along with hyphal forms of Candida (Figure 1). The budding yeasts were phagocytized by the neutrophils (Figure 2). Aerobic blood culture showed growth of C. tropicalis and C. parapsilosis following overnight incubation. The organism was observed to be growing in other body fluids as well, including sputum and urine. The patient was aggressively treated with fluconazole and later with amphotericin B; however, he died due to multiple organ failure. 

Candidemia is associated with high crude mortality rates, ranging from 30% to 81% [4]. The most common pathogen is C. albicans, which accounts for 70% of fungemia cases. Nonetheless, the results of a retrospective study conducted at a tertiary care center in Northern India showed that most episodes were cause by species other than C. albicans, the most frequent of which was C. tropicalis, followed by C. albicans (21.5%) and C. parapsilosis (20%). In the presented case C. tropicalis and C. parapsilosis were noted. Various fungal infections have been diagnosed via direct detection of fungi in peripheral blood smears [5]. In addition, leukoagglutination in a smear has been described as the actual host response to fungal invasion. Observation of such should prompt the laboratory hematologist to look for the presence of hyphae and yeast forms of Candida [5]. Whereas intra-/extraleukocytic Candida— both budding yeast forms—and hyphal forms were seen in the presented case’s blood smear, leukoagglutination was not. 

Treatment and prevention of candidemia has many challenges, including correct species identification and appropriate treatment, because different species are susceptible to different antifungal drugs. In contrast to other IFIs, such as invasive aspergillosis,[6] mortality associated with invasive candidiasis has not decreased significantly during the previous decade, despite the introduction of new classes of antifungal agents [7]. In addition, such infections are associated with prolonged hospitalization and treatment cost, constituting an enormous financial burden. In conclusion, we think that careful observation of peripheral blood smears is important for the early detection of fungal infection. 

**Conflict of Interest Statement**

The authors have no conflicts of interest, including specific financial interests, relationships, and/or affiliations, relevant to the subject matter or materials included.

## Figures and Tables

**Figure 1:**
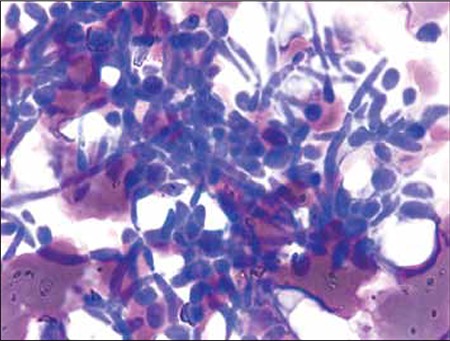
Extra-leukocytic yeast and hyphal forms of candida.Giemsa 1000X

**Figure 2:**
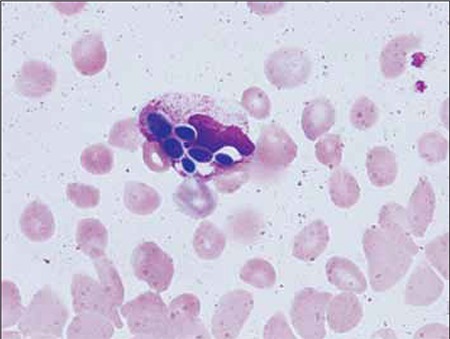
Phagocytosed candida in blood
